# Adult healthcare is associated with more emergency healthcare for young people with life-limiting conditions

**DOI:** 10.1038/s41390-022-01975-3

**Published:** 2022-02-12

**Authors:** Stuart Jarvis, Kate Flemming, Gerry Richardson, Lorna Fraser

**Affiliations:** 1 Martin House Research Centre, University of York, York, UK; 2 Department of Health Sciences, University of York, York, UK; 3 Centre for Health Economics, University of York, York, UK

## Abstract

**Background:**

Children with life-limiting conditions receive specialist paediatric care in childhood, but the transition to adult care during adolescence. There are concerns about transition, including a lack of continuity in care and that it may lead to increases in emergency hospital visits.

**Methods:**

A retrospective cohort was constructed from routinely collected primary and hospital care records for young people aged 12–23 years in England with (i) life-limiting conditions, (ii) diabetes or (iii) no long-term conditions. Transition point was estimated from the data and emergency inpatient admissions and Emergency Department visits per person-year compared for paediatric and adult care using random intercept Poisson regressions.

**Results:**

Young people with life-limiting conditions had 29% (95% CI: 14–46%) more emergency inpatient admissions and 24% (95% CI: 12–38%) more Emergency Department visits in adult care than in paediatric care. There were no significant differences associated with the transition for young people in the diabetes or no long-term conditions groups.

**Conclusions:**

The transition from paediatric to adult healthcare is associated with an increase in emergency hospital visits for young people with life-limiting conditions, but not for young people with diabetes or no long-term conditions. There may be scope to improve the transition for young people with life-limiting conditions.

**Impact:**

- There is evidence for increases in emergency hospital visits when young people with life-limiting conditions transition to adult healthcare.
- These changes are not observed for comparator groups - young people with diabetes and young people with no known long-term conditions, suggesting they are not due to other transitions happening at similar ages.
- Greater sensitivity to changes at transition is achieved through estimation of the transition point from the data, reducing misclassification bias.

## Introduction

There are many young people with life-limiting conditions—conditions that either shorten life, such as Duchenne Muscular Dystrophy, or conditions that threaten to shorten life, but may be cured, such as cancer^1^ —with ~86,000 in the United Kingdom and at least 500,000 in the United States.^2,3^

In childhood, care is normally led by a paediatric specialist. In the late teenage years, care transitions to adult services.^4,5^ Adult services are often coordinated in primary rather than secondary or tertiary care, but these providers can lack expertise and training in life-limiting conditions, despite efforts at improvement.^6^ Transition can seem abrupt,^5,7^ varying between health conditions and with availability and remit of local services.^5,8,9^ There is often no equivalent adult service.^10^ There can be a lack of follow-up, gaps in care and a lack of standardised transition.^11–13^

Any increase in emergency care at transition has implications for health service costs and may cause emotional trauma for young people and their families.^14–19^ Existing evidence on healthcare use at transition shows mixed findings,^20^ mainly from small, unrepresentative studies or larger studies with a simple age-based classification of transition.^21^ Transition age can vary widely,^5,8,9^ so these studies risk misclassification bias, with potential underestimation of transition effects.^22–24^

Transition can be managed well; research on transition for diabetes has suggested improved transition pathways.^25–36^ Diabetes, therefore, makes a good comparison group to life-limiting conditions to understand what changes might be expected under a well-established transition process. There are also other transitions at similar ages, such as changes in education and employment and increases in risk-taking behaviours that could affect emergency hospital use. Young people with no long-term health care conditions—and, therefore, no meaningful healthcare transition—are therefore another relevant comparison group.

This study aims to establish whether there is an increase in emergency inpatient admissions and Emergency Department visits when children with life-limiting conditions transition to adult healthcare using a nationally representative dataset.

## Methods

### Patient and public involvement

The Martin House Research Centre Family Advisory Board was consulted (the Board is one key part of the Centre’s PPI strategy^37^ and comprises parents and carers who either have or had children with life-limiting conditions). Transition experiences of families of children with life-limiting illness influenced the choice of comparator groups and informed the development of methods for estimating transition point and the final choice of approach.^22^ Their insights aided interpretation, as set out in the Discussion.

### Datasets

Nationally representative linked primary and secondary healthcare data from the Clinical Practice Research Datalink (CPRD, data from a sample of primary care providers in England) were used. CPRD identified all individuals in the CPRD ‘GOLD’ dataset aged 12–23 years at any point from 1 January 2000 to 31 December 2018. All records, while aged 0–23 years, were requested along with linked, Hospital Episode Statistics Admitted Patient Care (2000–2018), Outpatient (2000–2018) and Emergency Department (2007–2018) datasets. The datasets were linked by CPRD using National Health Service number, sex, date of birth and postcode.^38^

The study falls under ethical approval (ref: 05/MRE04/87) for observational research CPRD data approved by its Independent Scientific Advisory Committee (ISAC). ISAC approval was gained (protocol ref: 19_215R).

### Data management

#### Population of interest

Three groups of young people were of interest, as set out above: (i) those with life-limiting conditions, (ii) those with diabetes, and (iii) those with no known long-term conditions. Group membership depended on diagnoses in primary care, inpatient and outpatient records while aged 12–23 years:

1. Young people were assigned to the life-limiting conditions group if any diagnosis in the HES records matched a previously developed International Classification of Diseases, 10th Edition^39^ (ICD-10) coding framework^40^ or if any diagnosis in primary care records matched a Read coding framework^41^ derived from the ICD-10 coding framework.
2. Young people were assigned to the diabetes group, if not in the life-limiting conditions group and if any diagnosis in the HES or primary care records matched ICD-10 or Read codes for diabetes, derived from a previously developed list of chronic conditions diagnoses^42^ (see also Supplementary Material S1).
3. Young people were assigned to the no long-term conditions group if not in the other groups and if they had no matches in HES or primary care records against previously developed coding frameworks that identified health conditions likely (i.e. in more than 50% of cases) to require follow-up (hospital admissions, outpatient visits, medications) for more than 1 year.^42,43^

**Fig. 1:**
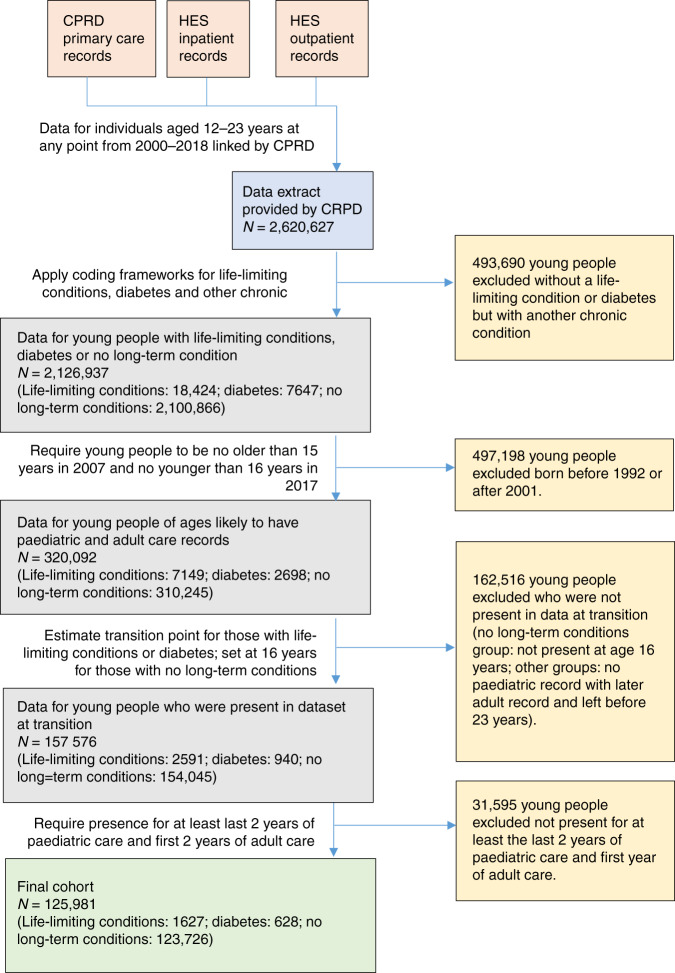
Cohort construction. Datasets held by CPRD are shown in pink; the final dataset provided to the authors by CPRD if shown in blue; grey boxes indicate processing steps; yellow boxes indicate exclusions and green boxes indicate the final data used in analyses. Arrows show data flows.

Young people not assigned to a group—i.e. those with a chronic condition other than diabetes or one of the life-limiting conditions—were excluded (Fig. 1).

#### Identification of transition point

**Fig. 2:**
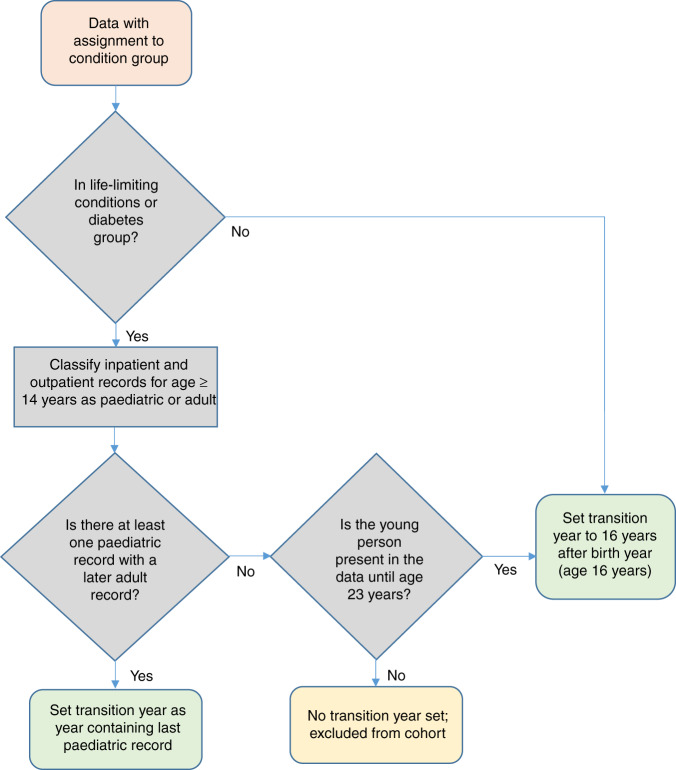
Flow chart of estimation of the transition point from the data. Pink box shows source data for the process; grey boxes show decisions and intermediate processing steps; green boxes show final outcomes for included data and yellow boxes show final outcomes for excluded data.

Transition points from paediatric to adult healthcare were estimated from the data for the life-limiting condition and diabetes groups, using a previously developed method.^22^ Transition point was identified by first classifying inpatient and outpatient records from age 14 years onwards for young people in the life-limiting conditions or diabetes groups as paediatric or adult based on treatment and consultant main speciality codes (Supplementary Material S2) and then applying rules to define transition point from these records (Fig. 2). Transition prior to 14 years was considered unrealistic, so records before 14 years were not considered.^5,6^

For young people in the life-limiting condition or diabetes groups, year of transition from children’s to adult care was set as the year containing the last paediatric record, as long as there was an adult record after the last recorded paediatric record.^22^ Those who died or left the dataset before age 23 years without an adult record after the last paediatric record were considered not to have undergone transition and were excluded from analysis (Figs. 1 and 2). Those who reached age 23 years without an adult record after the last paediatric record, due to a lack of secondary care records, were assigned transition at age 16. A sensitivity analysis set transition for all young people with no transition point estimated from the data to age 16 years (Supplementary Material S3).

For the no long-term condition group, transition year was set as 16 years after the year of birth (i.e. age 16 years).

Years before the estimated year of transition were considered paediatric healthcare. The year of transition and later years were considered adult healthcare.

An a priori decision was made to require presence for the last two years of paediatric care and the first two years of adult care (sensitivity analyses explored variations—see Supplementary Material S3). This meant anyone in the no long-term conditions group born after 2001 was excluded (as they were no older than 16 years at the study end in 2018 and so did not have two years of adult data). For consistency between groups, all young people born after 2001 were excluded.

Young people born before 1992 were excluded as they were unlikely to have records classified as paediatric (most paediatric specialities were introduced in 2007—anyone born before 1992 was already aged 16 years by 2007).

#### Cohort identification

A retrospective cohort was constructed (Fig. 1) including all young people who satisfied all the following criteria:

1. Were in the life-limiting conditions, diabetes or no long-term conditions groups.
2. Were born no earlier than 1992 and no later than 2001.
3. Had a transition point estimated.
4. Were present in the CPRD data for at least two years of paediatric care and two years of adult care.

#### Demographic data

Sex, year of birth, and deprivation category (derived from the 2015 Index of Multiple Deprivation based on last known address) were provided in CPRD data. Ethnic group was provided by CPRD as the group most commonly recorded in the linked HES data.

### Analyses

#### Description of cohort

Numbers of individuals in the cohort were summarised by condition group and demographics.

#### Transition age

Estimated age at transition was summarised for the life-limiting condition and diabetes groups

#### Emergency hospital visits

Numbers of emergency inpatient admissions and Emergency Department visits per person-year were summarised by age, condition group, sex and transition status (paediatric or adult care). Confidence intervals were estimated by bootstrapping (10,000 replications).

#### Statistical models

The two primary outcomes, number of inpatient admissions and Emergency Department visits were assessed in separate regression models.

##### Associations of numbers of emergency inpatient admissions with transition status

The outcomes were count data with repeated observations (one each year) clustered within individuals; a two-level (level 1: individual, level 2: year) random intercept Poisson regression was used.^44^ Over-dispersion, at least due to un-modelled differences between individuals, was accounted for by the random intercept.^44^ The independent variable of interest, transition status, was binary (1: adult; 0: paediatric). Other candidate variables were:

- Condition group (level 1) as healthcare use was expected to differ across these groups.
- Age (level 2) as healthcare use varies with age.^41,45^
- Sex (level 1) as healthcare use varies by sex.^41,45^
- Ethnic group (level 1) as healthcare use varies by ethnic group.^41^
- Deprivation category (level 1) as healthcare use varies by deprivation.^41,46^
- Year of birth (level 1) reflecting cohort effects if care practices changed over time.
  Interactions were also considered:
- Between age and sex and condition group, as healthcare use varies with age in different ways for males and females^45^ and may differ by condition group.
- Between transition status and condition group (it was expected that condition group would modify associations between transition and emergency hospital visits).

Reduction of the Bayesian Information Criterion by more than 3 was grounds for retention of variables and interactions.^47^

Time at risk (when present in the CPRD data and not a hospital inpatient) was included.

##### Associations of numbers of Emergency Department visits with transition status

A random intercept Poisson regression was used. Methods were the same as for emergency inpatient admissions, with the same candidate covariates and interactions.

#### Population-level estimates

**Fig. 3:**
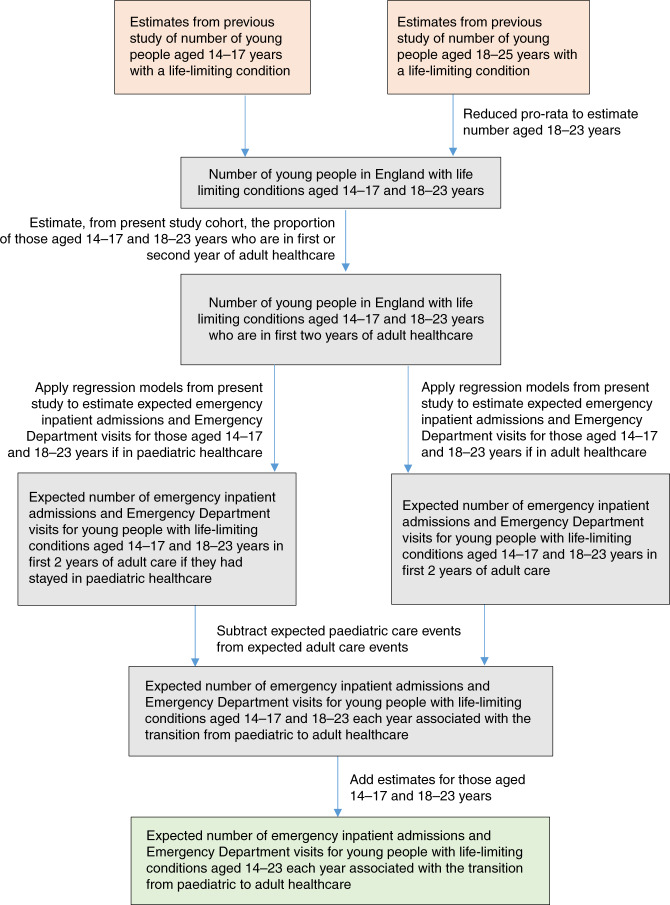
Construction of population-level estimates. Estimates of numbers additional emergency inpatient admissions and Emergency Department visits associated with a transition from paediatric to adult healthcare were combined with national estimates of young people aged 14–23 years with life-limiting conditions.

Estimates were made of changes in emergency inpatient admissions and Emergency Department visits associated with the transition for all young people with life-limiting conditions in England (Fig. 3). Numbers of young people aged 14–17 and 18–23 years with life-limiting conditions in England were estimated from 2017 (the most recently available) figures from a previously published full-population study.^48^ The proportion of young people aged 14–17 and 18–23 years in the present study data who were in the first two years of adult healthcare was calculated using estimated transition points, to give the number of young people aged 14–17 and 18–23 years in England in the first two years of adult healthcare (matching the period analysed in this study). Regression models from the present study were used to calculate expected numbers of emergency inpatient admissions and Emergency Department visits for members of the study cohort aged 14–17 and 18–23 years within this group if (a) they had remained in paediatric healthcare and (b) had transitioned to adult healthcare. These estimates for events per person per year were then multiplied by the number of young people nationally aged 14–17 and 18–23 years and in the first two years of adult care. The difference between these estimates was the expected difference in emergency hospital visits associated with the transition at the population level.

Confidence intervals in the final estimates were based on confidence intervals for the estimates from the regression models as the uncertainty from these models dominated other uncertainties.

## Results

### Cohort summary

There were 125,981 individuals in the final cohort (Fig. 1). There were more males than females in all three groups (Fig. 1 and Table 1). Comparisons between ethnic groups were hampered by large numbers of missing data in the no long-term conditions group (>50%). Less deprived groups were over-represented in the cohort as a whole (Table 1) but the distribution in the life-limiting conditions group was more even, with ~20% in each of the five deprivation categories.

**Table 1:** Cohort characteristics.

	Life-limiting conditions group	Diabetes group	No long-term conditions group	Full cohort
All in group	1627	628	123,726	125,981
Sex				
Males	920^a^ (56%)	350^a^ (56%)	66,020^a^ (53%)	67,290^a^ (53%)
Females	710^a^ (44%)	280^a^ (44%)	57,710^a^ (47%)	58,690^a^ (47%)
Unknown	≤10 (≤1%)	≤10 (≤1%)	≤10 (≤1%)	≤10 (≤1%)
Ethnic group				
Bangladeshi	≤10 (≤1%)	≤10 (≤2%)	153 (<1%)	162 (<1%)
Black	54 (3%)	≤10 (1%)	1334 (1%)	1396 (1%)
Indian	16 (1%)	≤10 (1%)	629 (1%)	649 (1%)
Pakistani	32 (2%)	≤10 (≤2%)	573 (<1%)	615 (1%)
White	1400 (86%)	573 (2%)	50,444 (41%)	52,417 (42%)
Mixed and other	64 (4%)	189 (91%)	2553 (<1%)	2635 (2%)
Unknown	50^a^ (3%)	10^a^ (≤2%)	68,040 (54%)	68,107 (54%)
Deprivation category				
1 (least deprived)	340^a^ (21%)	150^a^ (23%)	34,158 (28%)	34,644 (27%)
2	320^a^ (19%)	130^a^ (21%)	25,738 (21%)	26,183 (21%)
3	320^a^ (19%)	110^a^ (18%)	24,107 (19%)	24,532 (19%)
4	320^a^ (20%)	140^a^ (22%)	20,651 (17%)	21,106 (17%)
5 (most deprived)	340^a^ (21%)	110^a^ (17%)	18,983 (15%)	19,426 (15%)
Unknown	≤10 (≤1%)	≤10 (≤2%)	90^a^ (<1%)	90 (<1%)

Demographics are described for each of the condition groups and for the whole cohort.

Percentages are within the condition group.

^a^Indicates numbers rounded to the nearest 10 to prevent disclosure of censored numbers (≤10) in other cells.

### Transition age

**Fig. 4:**
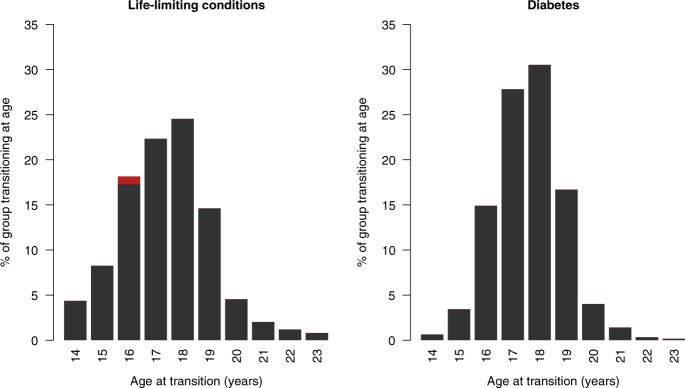
Distribution of transition ages in the life-limiting conditions and diabetes groups. Red sections on the bar for age 16 years indicates group members assigned this transition age due to lacking both a paediatric and an adult record (there were no such members in the diabetes group).

The most common transition age for diabetes and life-limiting conditions was 18 years, but the distribution was more closely grouped around 18 years for diabetes (Fig. 4). 1% of those with life-limiting conditions had transition assigned to age 16 years due to a lack of paediatric and adult records; all in the diabetes group had transition estimated from the data.

### Emergency hospital visits

#### Emergency inpatient admissions

The life-limiting conditions and diabetes groups had the highest rates of emergency admissions, at 0.28 (95% CI 0.26–0.31) and 0.26 (95% CI: 0.23–0.29) per person-year, respectively, (Table 2) and did not differ significantly (difference 0.02, 95% CI: −0.01–0.06). The no long-term conditions group had a lower rate, 0.0162 (95% CI: 0.0160–0.0164) per person-year.

**Table 2:** Emergency hospital visits by conditions group, sex and transition status.

Group	Emergency inpatient admissions per 100 person years (95% CI)			Emergency Department visits per 100 person years (95% CI)		
	Life-limiting conditions	Diabetes	No long-term conditions	Life-limiting conditions	Diabetes	No long-term conditions
All	28 (25–31)	26 (23–29)	1.62 (1.60–1.64)	55 (51–58)	65 (59–70)	19.7 (19.6–19.8)
Males	25 (22–28)	21 (18–23)	1.78 (1.76–1.81)	50 (46–53)	60 (52–66)	22.9 (22.7–23.0)
Females	32 (27–37)	33 (28–38)	1.43 (1.40–1.46)	61 (54–68)	71 (64–79)	16.0 (15.8–16.1)
Paediatric care	27 (24–30)	27 (24–30)	16.2 (1.60–1.65)	51 (48–55)	64 (59–69)	19.6 (19.5–19.7)
Adult care	33 (29–37)	24 (20–28)	1.61 (1.55–1.67)	65 (58–71)	67 (57–76)	20.2 (20.0–20.5)

For ease of comparison of rare events (emergency inpatient admissions for those with no long-term conditions) these are expressed by 100 person years.

**Fig. 5:**
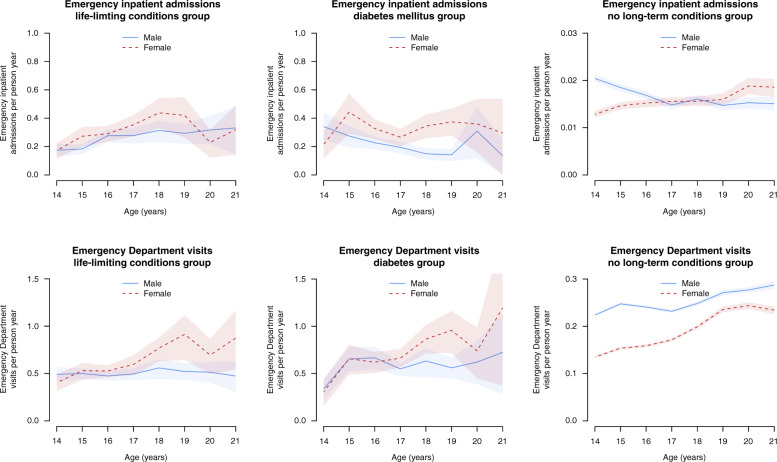
Numbers of emergency healthcare events by age and sex. Numbers of emergency inpatient admissions and Emergency Department visits recorded per person-year in the cohort, split by age and sex, with 95% confidence intervals (shaded areas) estimated by bootstrapping.

Females had more emergency inpatient admissions than males in the life-limiting conditions and diabetes groups (Table 2 and Fig. 5), by 0.07 (95% CI: 0.01–0.12) in the life-limiting conditions group and 0.13 (95% CI: 0.07–0.18) in the diabetes group per person year. For no long-term conditions, males had 0.0040 (95% CI: 0.0031–0.0035) more admissions per person-year than females. Trends by age differed between sexes (Fig. 5) with increases with age for both males and females in the life-limiting conditions group (although mostly within 95% confidence intervals); decreases for males with diabetes up to age 19 years and no clear change for females; and increases for females and decreases for males in the no long-term conditions group.

**Fig. 6:**
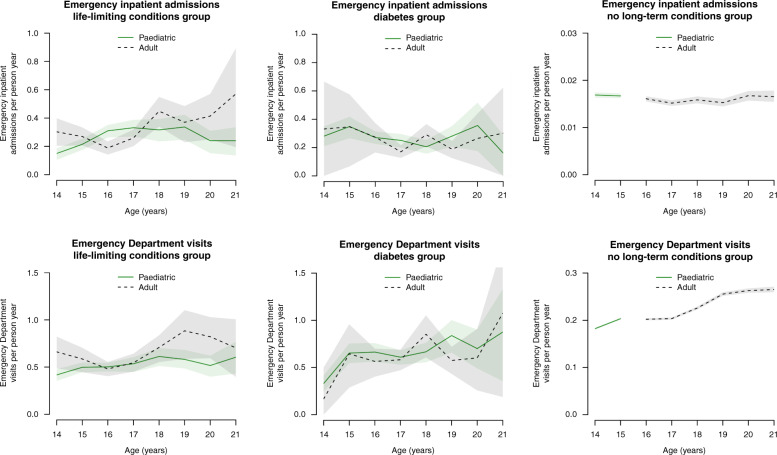
Numbers of emergency healthcare events by age and transition status. Numbers of emergency inpatient admissions and Emergency Department visits recorded per person-year in the cohort, split by age and transition status, with 95% confidence intervals (shaded areas) estimated by bootstrapping.

In the life-limiting conditions group, those in adult care had 0.06 (95% CI: 0.03–0.09) more emergency admissions per person-year than those in paediatric care (Table 2 and Fig. 6). There were no significant differences for the other groups. Differences varied by age in the life-limiting conditions group (Fig. 6), with those aged 16 years in adult care having fewer emergency admissions than those in paediatric care at the same age; at most other ages point estimates were higher in adult care than paediatric care.

#### Emergency Department visits

The life-limiting conditions and diabetes groups had the highest rates of Emergency Department visits, at 0.55 (95% CI: 0.51–0.58) and 0.65 (95% CI: 0.59–0.70) per person-year respectively (Table 2); visits were higher in the diabetes group (by 0.10, 95% CI: 0.03–0.16). The no long-term conditions group had fewer visits: 0.197 (95% CI: 0.196–0.198) per person-year.

Females with life-limiting conditions and diabetes had more visits per person-year than males (Table 2 and Fig. 5) by, respectively, 0.11 (95% CI: 0.03–0.19) and 0.11 (95% CI: 0.01–0.22). Males with no long-term conditions (Table 2) had 0.069 (95% CI: 0.066–0.071) more visits than females. Trends by age differed between sexes (Fig. 5) with greater increases with increasing age for females than for males.

In the life-limiting conditions group, those in adult care had 0.13 (95% CI: 0.08–0.18) more visits per person-year than those in paediatric care (Table 2 and Fig. 6). There were no significant differences for the diabetes group. In the no long-term conditions group, those in adult care had 0.006 (95% CI: 0.004–0.008) more visits than those in paediatric care. Differences varied by age in the life-limiting conditions group (Fig. 6), with those aged 16–17 years in adult care having little difference in point estimates for visits compared to those in paediatric care at the same age, although at most other ages point estimates were higher for the group in adult care. There were no clear differences by transition status for the diabetes group. For the no long-term conditions group, those in adult care had more visits than those in paediatric care, but were also older.

### Statistical models

The final regression models included transition status (adult or paediatric care), age in year, sex, ethnic group, deprivation category, condition group, an interaction between transition status and condition group and an interaction between condition group, age and sex (Table 3). Sensitivity analyses excluding deprivation category and ethnic group and including the year of birth are presented in Supplementary Material S3.

**Table 3:** Regression models for emergency healthcare events.

	Emergency inpatient admissions				Accident and Emergency Department visits			
	Incidence rate ratio	95% confidence interval		*P* value	Incidence rate ratio	95% confidence interval		*P* value
Age (per year of age)	0.90	0.88	0.93	<0.01	1.02	1.01	1.03	<0.01
Sex								
Male	1 (ref)				1 (ref)			
Female	0.06	0.03	0.12	<0.01	0.23	0.18	0.28	<0.01
Ethnic group								
Bangladeshi	1.32	0.86	2.02	0.20	0.81	0.60	1.08	0.15
Black African	1.52	1.12	2.05	0.01	1.00	0.83	1.21	0.98
Black Caribbean	1.07	0.69	1.64	0.77	0.94	0.79	1.12	0.49
Black Other	0.88	0.65	1.20	0.41	0.92	0.80	1.05	0.21
Chinese	1.07	0.69	1.67	0.76	0.58	0.45	0.74	<0.01
Indian	0.77	0.60	0.99	0.04	0.64	0.57	0.72	<0.01
Mixed	1.07	0.88	1.30	0.48	1.11	0.99	1.25	0.08
Other Asian	1.02	0.78	1.34	0.88	0.77	0.67	0.89	<0.01
Other	0.86	0.72	1.03	0.11	0.88	0.80	0.96	0.01
Pakistani	0.77	0.58	1.02	0.07	0.79	0.70	0.90	<0.01
White	1 (ref)				1 (ref)			
Unknown	0.03	0.03	0.04	<0.01	0.48	0.47	0.49	<0.01
Deprivation category								
1 (least deprived)	1 (ref)				1 (ref)			
2	0.92	0.85	0.99	0.03	1.03	1.00	1.06	0.02
3	0.95	0.88	1.02	0.17	1.11	1.08	1.14	<0.01
4	1.06	0.98	1.14	0.14	1.21	1.18	1.25	<0.01
5 (most deprived)	1.06	0.97	1.15	0.19	1.31	1.27	1.35	<0.01
Condition group								
No long-term condition	1 (ref)				1 (ref)			
Diabetes	6.63	0.96	45.61	0.06	2.16	0.57	8.27	0.26
Life-limiting condition	1.62	0.50	5.26	0.42	2.36	1.09	5.11	0.03
Transition status								
Paediatric care	1 (ref)				1 (ref)			
Adult care	1.00	0.94	1.06	1.00	1.01	0.99	1.02	0.46
Sex × age interaction^a^								
Female (per year of age)	1.18	1.13	1.24	<0.01	1.07	1.06	1.09	<0.01
Condition group × age interaction^a^								
Diabetes (per year of age)	1.00	0.89	1.13	0.95	0.99	0.91	1.06	0.71
Life-limiting conditions (per year of age)	1.10	1.03	1.18	0.01	0.97	0.92	1.02	0.26
Condition group × sex × age interaction^a^								
Diabetes and female (per year of age)	0.99	0.83	1.18	0.91	1.11	0.99	1.25	0.08
Life-limiting conditions and female (per year of age)	0.85	0.76	0.95	0.01	1.03	0.96	1.11	0.34
Condition group × transition status interaction^a^								
Diabetes and adult care	0.83	0.67	1.01	0.07	0.93	0.82	1.06	0.30
Life-limiting condition and adult care	1.29	1.13	1.48	<0.01	1.24	1.12	1.37	<0.01

Regression model incidence rate ratio for two-level Poisson regressions on the numbers of emergency inpatient admissions and number of Emergency Department visits per cohort member per year.

^a^Indicates there are omitted combinations of interactions (reference groups with an incident rate ratio 1).

#### Emergency inpatient admissions

Young people in adult care in the life-limiting conditions group had 29% (95% CI: 14–46%) more emergency inpatient admissions than those in paediatric care. There were no significant differences associated with the transition for the other groups (Table 4).

**Table 4:** Differences in emergency healthcare use between adult and paediatric care.

Group	Emergency inpatient admissions				Accident and Emergency Department visits			
	Incidence rate ratio (adult compared to paediatric care)	95% confidence interval		*P* value	Incidence rate ratio (adult compared to paediatric care)	95% confidence interval		*P* value
Life-limiting conditions	1.29	1.14	1.46	<0.01	1.24	1.12	1.38	<0.01
Diabetes	0.83	0.68	1.00	0.06	0.94	0.83	1.07	0.35
No long-term conditions	1.00	0.94	1.06	1.00	1.01	0.99	1.02	0.46

Combined incidence rate ratios (taking account of interactions) for emergency inpatient admissions and Emergency Department visits.

#### Emergency Department visits

Young people in the life-limiting conditions group in adult care had 24% (95% CI: 12–38%) more Emergency Department visits than those in paediatric care. There were no significant differences associated with the transition for the other groups (Table 4). A gradient was observed with deprivation for Emergency Department visits, with the most deprived groups having 31% (95% CI: 27–35%) more visits than the least deprived group.

### Population-level estimates

For young people with life-limiting conditions aged 14–23 years and in their first two years of adult care, the regression models predict an extra 753 (95% CI: 550–1031) emergency inpatient admissions and an extra 1201 (95% CI: 876–1630) Emergency Department visits each year compared to remaining in paediatric care (see also Supplementary Material S4 for splits by 14–17 and 18–23 years age groups).

## Discussion

This study showed an increase in unplanned hospital visits associated with the transition from paediatric to adult healthcare for young people with life-limiting conditions, of 29% (95% CI: 14–46%) for emergency inpatient admissions and 24% (95% CI: 12–38%) for Emergency Department visits. No significant change in unplanned hospital visits was found for the diabetes and no long-term conditions groups.

The changes associated with the transition for the life-limiting conditions group are equivalent to an extra 753 (95% CI: 550–1031) emergency inpatient admissions and 1201 (95% CI: 876–1630) Emergency Department visits each year.

### Variations by condition group

Findings for the condition groups differed greatly, with associations found between transition and unplanned hospital visits for the life-limiting conditions groups, but not for the diabetes and no long-term conditions groups.

For the no long-term conditions group, there should be no meaningful healthcare transition —hospital visits should be mainly ad-hoc and so it would be usual to see a different healthcare practitioner in a different department for each visit. There is no additional discontinuity in care around age 16 years—consistent with the observed lack of an association between transition status and emergency hospital visits.

No change at transition was found for the diabetes group. A previous review for diabetes found mixed evidence on hospital use around transition, from increases to no change.^35^ Established transition pathways may minimise impact.^25,26,30,33,34^ Young people with diabetes are a far more homogenous group than young people with life-limiting conditions, with similar treatments and complications. Many primary care practitioners would have the ongoing experience of caring for young people with diabetes. They have fewer complex needs and less need for associated services, such as physiotherapy, that transition could disrupt. Transition ages were more tightly clustered for the diabetes group than for the life-limiting conditions group, which may reflect greater uniformity in processes or needs. The present study shows that transition from paediatric to adult services does not necessarily have to lead to an increase in emergency hospital visits. There may be scope to reduce unplanned hospital visits after transition for the life-limiting conditions group by learning lessons from the transition processes for diabetes, particularly around greater care continuity being associated with reduced risk of hospitalisation.^31,33^ For diabetes, unlike for some life-limiting conditions, there are adult services closely equivalent to paediatric services. Continuity and knowledge in primary care have been cited as helpful by members of the Martin House Research Centre Family Advisory Board and in previous studies.^41,49^

For young people with life-limiting conditions, the findings are consistent with some previous studies that found evidence of increases in inpatient admissions at transition.^20,50–53^ Studies finding fewer inpatient admissions post-transition were clustered in Canada^54–57^ and looked at all admissions, not only emergency inpatient admissions. They also, unlike the present study, did not estimate transition point from the data. One study looking at emergency inpatient admissions found conflicting evidence for males and females, but across a narrow range of blood conditions.^58^ The findings of increased Emergency Department visits associated with the transition are consistent with the majority of previous studies looking at this outcome.^20,55,56,59,60^ The findings were consistent with the experiences of the Martin House Research Centre Family Advisory Board, who cited a switch to more reactive rather than preventive care after transition, poorer condition management, inconsistency in staff seen in primary care and subsequent lack of understanding and trust as possible reasons for increases in emergency hospital visits, factors backed up by other studies.^7,13,31,41^

### Implications of findings

The models demonstrate associations, not causality, but the comparator groups of diabetes and no long-term conditions exclude some other possible explanations such as inevitable changes in healthcare-seeking behaviours around transition ages or changes related to risk-taking behaviours. Increases in unplanned hospital visits associated with the transition will have emotional and financial impacts on young people with life-limiting conditions and their families.^14–19,61^ Emergency inpatient admissions, at least, may also be indicative of a deteriorating or poorly managed condition, with longer-term implications for ongoing care needs and quality of life. The population-level estimates put the observed changes at transition in perspective. They look only at the first two years of adult care (sensitivity analyses suggest that associations persist for longer) so represent a conservative estimate.

There is a clear contrast between the life-limiting conditions and diabetes groups in the present study. Both groups of young people undergo a meaningful transition from paediatric to adult healthcare, with changes in care providers and an expectation for the young person to play a greater role in condition management.^5–13,25–36^ However, in the present study, increases in emergency healthcare use are only observed for the life-limiting conditions group. Transition for young people with diabetes is not free of problems,^35^ but there is generally a defined transition process^62^ and adult services have broadly similar provision to child services, albeit with often less frequent contact, and diabetes is commonly managed in primary care.^63^ While there are concerns about discontinuity, young people with diabetes are also focused on the challenges of taking greater responsibility for their care and the interaction with other behavioural changes and life events at similar ages—changes in education and in risk-taking behaviours.^35^ This is in contrast to services for young people with life-limiting conditions where there may not be an equivalent adult service,^10^ there may be little expertise in particular conditions among primary can practitioners^6^ and transition processes can vary by condition.^5,8,9^

The results for diabetes show that the transition from children’s to adult healthcare does not necessarily have to be associated with an increase in emergency hospital care. However, it does not follow that simply copying the diabetes transition would improve care for young people with life-limiting conditions. The latter group have more diverse healthcare needs which may be less easily met by primary care generalists and are likely to require at least partly condition-specific transition programmes.

### Strengths and limitations

This study has a number of strengths. Unlike many previous studies, it uses nationally representative routinely collected healthcare data, so reduces the risk of bias from—for example—small groups within a single clinic. It also estimates the transition point from the data, increasing sensitivity to detect changes in healthcare use associated with the transition.^22^ It makes use of comparison groups, suggesting observed changes are not due to non-healthcare transitions occurring at similar ages. Sensitivity analyses were used to test impacts key assumptions and analysis decisions, particularly around years of data to include in the regressions.

There are also limitations. There may be power issues for the relatively small diabetes group. The study sample size was chosen based on simulations of a 20% change in outcomes at transition, so while any changes at transition for the diabetes group can be expected to be less than 20%, smaller changes may be present that this study was not powered to detect. There were many missing data for ethnic group, but mostly for the no long-term conditions group, with more than 50% missing. Sensitivity analyses suggest this had little effect on estimates. There will also likely be some individuals for whom transition point was misidentified or for whom transition was a multi-year process. These issues are however likely to be fewer and smaller than in other studies that used a simple age cut-off to assign transition status. Finally, healthcare transitions are not the only transitions taking place during the years of data included in the study. Other transitions, particularly in education or employment can also happen at similar ages and impacts may differ between condition groups. As noted by the Martin House Research Centre Family Advisory Board, young people with life-limiting conditions may have attended specialist schools with regular access to nurses, so school transitions may be more impactful on health outcomes for this group than for the others.

### Future research

The findings of this study suggest that there is an increase in emergency hospital visits in the first two years of adult healthcare. Further studies are needed to understand this, looking—through qualitative research—at the experiences of young people with life-limiting conditions as they transition.

There is also a need for research into other aspects of healthcare at transition, including other measures of secondary care use (e.g. length of stay, bed days per year) and measures of primary care use, such as GP contacts. The relationships, if any, between primary and secondary care use before and after transition should also be explored. Costs, to both healthcare providers and young people and their families, should also be assessed to help understand the scope for cost-effective changes in care.

Understanding experiences and needs, as well as a full picture of healthcare use, across the transition, will help to focus future research on possible areas of intervention. Any interventions should be rigorously assessed for impact.

## Conclusion

The transition from paediatric to adult healthcare is associated with an increase in emergency hospital visits for young people with life-limiting conditions. Such an increase is not seen for young people with diabetes or no long-term conditions.

## Supplementary information

Record checklist

Supplementary Materials
