# Supplementary material for: Adult healthcare is associated with more emergency healthcare for young people with life-limiting conditions
Source: Pediatr Res. 2022 Feb 12;92(5):1458–69. doi: 10.1038/s41390-022-01975-3 (PMC9700517; doi:10.1038/s41390-022-01975-3)
Supplement: Supplementary file 2 — Supplementary Materials [file 41390_2022_1975_MOESM2_ESM.pdf]

Supplementary material to:

Adult healthcare is associated with more emergency healthcare for young people with life-limiting conditions

Supplementary material S1: Coding frameworks used to assign individuals to condition groups

Table S1: ICD-10 and Read codes used to identify young people with diabetes. ICD-10 codes are shown as 3 digits and include all 4 digit codes beginning with these 3 digits.

ICD-10	Read
E10, E11, E12, E13, E14	Cyu2.00, C10..00, C10C.00, C10D.00, C10H.00, C102z00, C101z00, C103z00, C106z00, C100z00, C105z00, C10yz00, C107z00, C10zz00, C107.11, C102.00, C101.00, C103.00, C104z00, C106.12, C10<0.01, C105.00, C10y.00, C107.00, C106.13, C104.00, C10z.00, C107000, C105000, C10z000, C100000, C102000, C101000, C103000, C104000, C106000, C10y000, 66AI.00, 66AJ.00, 66AJz00, 66A5.00, 66o6.00, 66AV.00, 66o2.00, 66A4.00, 66o5.00, 66As.00, C108.11, C108J00, C108G00, C108900, C10E912, C100011, C108.00, C10E.12, C108800, C10E812, C108H00, C108F00, C10EF12, C108600, C10E612, C108E00, C10EE12, C108B00, C10E312, C108300, C108D00, C10ED12, C108C00, C10EC12, C108700, C10E712, C108500, C10E512, C109J11, C10FJ00, C109J00, C10FJ11, C108200, C10E212, C108100, C10E112, C108000, C10E012, C108A00, C10EA12, C10M.00, K01x100, C109E00, C109H00, C100112, C109700, C109G00, C109500, C109D00, C109A00, C109C00, C109B00, C109400, C109F00, C109300, C109200, C109100, C109000, C109900, C10N.00, C10N000, C10G.00, C10G000, C10E.00, C108.12, C10E800, C108812, C10P011, C10E900, C108912, C10EH00, C10EF00, C108F12, C10EP00, C10E600, C10EQ00, C10EE00, C108E12, C10EM00, C10EN00, C10EB00, C10E300, C10ED00, C10E200, C108212, C108J12, C10EJ00, C10E100, C108112, C10EG00, C10EL00, C10EK00, C10EC00, C108012, C10E000, C10E700, C108712, C108512, C10EA00, C108A12, C10F.00, C109.12, C10F700, C109712, C10P111, C109G12, C10FG00, C109E12, C10FE00, C10FQ00, C10F500, C109512, C10FR00, C10FD00, C109D12, C10FN00, C10FP00, C10FA00, C10F300, C109312, C10FC00, C109C12, C10F200, C109212, C10FH00, C109H12, C10F100, C109112, C10FF00, C109F12, C10FM00, C10FL00, C10FB00, C109B12, C10F000, C10F600, C109612, C10F400, C109412, C10F900, C109912, C108.13, C108811, C10E811, C10P000, C108911, C10E911, C108H11, C108F11, C10EP11, C10E611, C10EQ11, C108E11, C10EM11, C10EN11, C108B11, C10E311, C108311, C108D11, C108211, C108J11, C10E111, C10EL11, C10EC11, C108011, C10E011, C108711, C10E711, C108511, C10E511, C10EA11, C108A11, 66At000, C109.13, C10F.11, C109711, C10F711, C10P100, C109G11, C10FG11, C109E11, C10FE11, C10FQ11, C109511, C10F511, C109D11, C10FD11, C10FN11, C10FP11, C109A11, C10FA11, C10F311, C109C11, C10FC11, C109211, C10F211, C109H11, C10FH11, C109111, C10F111, C109F11, C10FF11, C10FL11, C109B11, C10FB11, C109011, C10F011, C10F611, C109611, C109411, C10F411, C10F911, C109911, C108z00

Supplementary material S2: Classification of records as adult or paediatric

Table S2: Classification of treatment and consultant main specialties as paediatric or adult.

Treatment Specialty	Paediatric	Adult	Unclassified
100 = General Surgery		Y	
101 = Urology		Y	
102 = Transplantation Surgery (Includes Renal And Liver Transplants, Excludes Cardiothoracic Transplantation)		Y	
103 = Breast Surgery (Includes Suspected Neoplasms, Cysts Etc, Does Not Include Cosmetic Surgery)		Y	
104 = Colorectal Surgery (Surgical Treatment Of Disorders Of The Lower Intestine - Colon, Anus And Rectum)		Y	
105 = Hepatobiliary & Pancreatic Surgery (Includes Liver Surgery But Excludes Liver Transplantation See Transplantation Surgery)		Y	
106 = Upper Gastrointestinal Surgery		Y	
107 = Vascular Surgery		Y	
108 = Spinal Surgery Service (From April 2013)		Y	
110 = Trauma & Orthopaedics			Y
120 = Ear, Nose And Throat (ENT)		Y	
130 = Ophthalmology		Y	
140 = Oral Surgery		Y	
141 = Restorative Dentistry (Endodontics, Periodontics And Prosthodontics)		Y	
142 = Paediatric Dentistry	Y		
143 = Orthodontics			Y
144 = Maxillo-Facial Surgery		Y	
150 = Neurosurgery		Y	
160 = Plastic Surgery		Y	
161 = Burns Care (Recognised Specialist Services Only - Includes 'Outreach' Facilities)		Y	
170 = Cardiothoracic Surgery (Where There Are No Separate Services For Cardiac And Thoracic Surgery)		Y	
171 = Paediatric Surgery	Y		
172 = Cardiac Surgery		Y	
173 = Thoracic Surgery		Y	
174 = Cardiothoracic Transplantation (Recognised Specialist Services Only - Includes 'Outreach' Facilities)		Y	
180 = Emergency (EMERGENCY DEPARTMENT)			Y
190 = Anaesthetics		Y	
191 = Pain Management (Complex Pain Disorders Requiring Diagnosis And Treatment By A Specialist Multi-Professional Team)		Y	
192 = Critical Care Medicine (Also Known As Intensive Care Medicine)		Y	

199 = Non-Uk Provider - Specialty Function Not Known, Treatment Mainly Surgical		Y	
211 = Paediatric Urology (From 2006-07)	Y		
212 = Paediatric Transplantation Surgery (From 2006-07)	Y		
213 = Paediatric Gastrointestinal Surgery (From 2006-07)	Y		
214 = Paediatric Trauma And Orthopaedics (From 2006-07)	Y		
215 = Paediatric Ear Nose And Throat (From 2006-07)	Y		
216 = Paediatric Ophthalmology (From 2006-07)	Y		
217 = Paediatric Maxillo-Facial Surgery (From 2006-07)	Y		
218 = Paediatric Neurosurgery (From 2006-07)	Y		
219 = Paediatric Plastic Surgery (From 2006-07)	Y		
220 = Paediatric Burns Care (From 2006-07)	Y		
221 = Paediatric Cardiac Surgery (From 2006-07)	Y		
222 = Paediatric Thoracic Surgery (From 2006-07)	Y		
223 = Paediatric Epilepsy (From April 2013)	Y		
241 = Paediatric Pain Management (From 2006-07)	Y		
242 = Paediatric Intensive Care (From 2006-07)	Y		
251 = Paediatric Gastroenterology (From 2006-07)	Y		
252 = Paediatric Endocrinology (From 2006-07)	Y		
253 = Paediatric Clinical Haetology (From 2006-07)	Y		
254 = Paediatric Audiological Medicine (From 2006-07)	Y		
255 = Paediatric Clinical Immunology And Allergy (From 2006-07)	Y		
256 = Paediatric Infectious Diseases (From 2006-07)	Y		
257 = Paediatric Dermatology (From 2006-07)	Y		
258 = Paediatric Respiratory Medicine (From 2006-07)	Y		
259 = Paediatric Nephrology (From 2006-07)	Y		
260 = Paediatric Medical Oncology (From 2006-07)	Y		
261 = Paediatric Metabolic Disease (From 2006-07)	Y		
262 = Paediatric Pheumalogy (From 2006-07)	Y		
263 = Paediatric Diabetic Medicine	Y		
264 = Paediatric Cystic Fibrosis	Y		
280 = Paediatric Interventional Radiology (From 2006-07)	Y		
290 = Community Paediatrics (From 2006-07)	Y		
291 = Paediatric Neuro-Disability (From 2006-07)	Y		
300 = General Medicine		Y	
301 = Gastroenterology		Y	
302 = Endocrinology		Y	
303 = Clinical Haematology		Y	
304 = Clinical Physiology (From 2008-09)		Y	
305 = Clinical Pharmacology		Y	
306 = Hepatology		Y	
307 = Diabetic Medicine		Y	
308 = Bone And Marrow Transplantation (Previously Part Of Clinical Haematology)		Y	
309 = Haemophilia (Previously Part Of Clinical Haematology)		Y	
310 = Audiological Medicine		Y	

311 = Clinical Genetics		Y	
313 = Clinical Immunology And Allergy		Y	
314 = Rehabilitation Service		Y	
315 = Palliative Medicine		Y	
316 = Clinical Immunology		Y	
317 = Allergy Service		Y	
318 = Intermediate Care		Y	
319 = Respite Care		Y	
320 = Cardiology		Y	
321 = Paediatric Cardiology	Y		
322 = Clinical Microbiology		Y	
323 = Spinal Injuries (From 2006-07)		Y	
324 = Anticoagulant Service		Y	
325 = Sport And Exercise Medicine		Y	
327 = Cardiac Rehabilitation		Y	
328 = Stroke Medicine		Y	
329 = Transient Ischaemic Attack		Y	
330 = Dermatology		Y	
331 = Congenital Heart Disease Service (From April 2013)		Y	
340 = Respiratory Medicine (Previously Known As Thoracic Medicine)		Y	
341 = Respiratory Physiology (Previously Known As Sleep Studies)		Y	
342 = Programmed Pulmonary Rehabilitation		Y	
343 = Adult Cystic Fibrosis Service		Y	
344 = Complex Specialised Rehabilitation Service (From April 2013)		Y	
345 = Specialist Rehabilitation Service (From April 2013)		Y	
346 = Local Specialist Rehabilitation Service (From April 2013)		Y	
350 = Infectious Diseases		Y	
352 = Tropical Medicine		Y	
360 = Genitourinary Medicine		Y	
361 = Nephrology		Y	
370 = Medical Oncology		Y	
371 = Nuclear Medicine (From 2008-09)		Y	
400 = Neurology		Y	
401 = Clinical Neurophysiology (From 2008-09)		Y	
410 = Rheumatology		Y	
420 = Paediatrics	Y		
421 = Paediatric Neurology	Y		
422 = Neonatology		Y	
424 = Well Babies (Care Given By The Mother/Substitute, With Nursing AdviceNeeded)		Y	
430 = Geriatric Medicine		Y	
450 = Dental Medicine Specialities		Y	
460 = Medical Ophthalmology		Y	
501 = Obstetrics		Y	
502 = Gynaecology		Y	
503 = Gynaecological Oncology		Y	

560 = Midwifery Service		Y	
650 = Physiotherapy (From 2006-07)			Y
651 = Occupational Therapy (From 2006-07)		Y	
652 = Speech And Language Therapy (From 2006-07)		Y	
653 = Podiatry (From 2006-07)		Y	
654 = Dietetics (From 2006-07)			Y
655 = Orthoptics (From 2006-07)		Y	
656 = Clinical Psychology (From 2006-07)			Y
657 = Prosthetics		Y	
658 = Orthotics		Y	
659 = Drama Therapy		Y	
660 = Art Therapy		Y	
661 = Music Therapy		Y	
662 = Optometry		Y	
663 = Podiatric Surgery (From April 2013)		Y	
700 = Learning Disability (Previously Known As Mental Handicap)		Y	
710 = Adult Mental Illness		Y	
711 = Child And Adolescent Psychiatry	Y		
712 = Forensic Psychiatry		Y	
713 = Psychotherapy		Y	
715 = Old Age Psychiatry		Y	
720 = Eating Disorders (From 2006-07)		Y	
721 = Addiction Services (From 2006-07)		Y	
722 = Liaison Psychiatry (From 2006-07)		Y	
723 = Psychiatric Intensive Care(From 2006-07)		Y	
724 = Perinatal Psychiatry (From 2006-07)		Y	
725 = Mental Health Recovery And Rehabilitation Service (From April 2013)		Y	
726 = Mental Health Dual Diagnosis Service (From April 2013)		Y	
727 = Dementia Assessment Service (From April 2013)		Y	
800 = Clinical Oncology (Previously Known As Radiotherapy)		Y	
811 = Interventional Radiology		Y	
812 = Diagnostic Imaging (From 2008-09)			Y
822 = Chemical Pathology		Y	
834 = Medical Virology		Y	
840 = Audiology (From 2008-09)		Y	
920 = Diabetic Education Service (From April 2013)			Y
**Consultant Main Specialty**	**Paediatric**	**Adult**	**Unclassified**
100 = General Surgery		Y	
101 = Urology		Y	
110 = Trauma And Orthopaedics		Y	
120 = Ear, Nose And Throat (Ent)		Y	
130 = Ophthalmology		Y	
140 = Oral Surgery		Y	
141 = Restorative Dentistry		Y	
142 = Paediatric Dentistry (Available From 1999-2000)		Y	
143 = Orthodontics		Y	

145 = Oral And Maxillo Facial Surgery (Available From 2004-05)		Y	
146 = Endodontics (Available From 2004-05)		Y	
147 = Periodontics		Y	
148 = Prosthodontics (Available From 2004-05)		Y	
149 = Surgical Dentistry (Available From 2004-05)		Y	
150 = Neurosurgery		Y	
160 = Plastic Surgery		Y	
170 = Cardiothoracic Surgery		Y	
171 = Paediatric Surgery	Y		
180 = Accident And Emergency (EMERGENCY DEPARTMENT)			Y
190 = Anaesthetics		Y	
191 = Pain Management (Available From 1998-99 To 2003-04)		Y	
192 = Critical Care Medicine (Available From 2004-05)		Y	
300 = General Medicine		Y	
301 = Gastroenterology		Y	
302 = Endocrinology		Y	
303 = Clinical Haematology		Y	
304 = Clinical Physiology		Y	
305 = Clinical Pharmacology		Y	
310 = Audiological Medicine		Y	
311 = Clinical Genetics		Y	
312 = Clinical Cytogenics And Molecular Genetics (Available From 1990-91)		Y	
313 = Clinical Immunology And Allergy (Available From 1991-92)		Y	
314 = Rehabilitation (Available From 1991-92)		Y	
315 = Palliative Medicine		Y	
320 = Cardiology		Y	
321 = Paediatric Cardiology (Available From 2004-05)	Y		
325 = Sport And Exercise Medicine		Y	
326 = Acute Internal Medicine		Y	
330 = Dermatology		Y	
340 = Respiratory Medicine (Also Known As Thoracic Medicine)		Y	
350 = Infectious Diseases		Y	
352 = Tropical Medicine (Available From 2004-05)		Y	
360 = Genito-Urinary Medicine		Y	
361 = Nephrology		Y	
370 = Medical Oncology		Y	
371 = Nuclear Medicine		Y	
400 = Neurology		Y	
401 = Clinical Neuro-Physiology		Y	
410 = Rheumatology		Y	
420 = Paediatrics	Y		
421 = Paediatric Neurology	Y		
430 = Geriatric Medicine		Y	
450 = Dental Medicine (Available From 1990-91)		Y	

451 = Special Care Dentistry		Y	
460 = Medical Ophthalmology (Available From 1993-94)		Y	
499 = Non-Uk Provider - Specialty Function Not Known, Treatment Mainly Medical		Y	
500 = Obstetrics And Gynaecology		Y	
501 = Obstetrics (Prior To 2004-05: Obstetrics For Patients Using A Hospital Bed Or Delivery Facilities)		Y	
502 = Gynaecology		Y	
504 = Community Sexual And Reproductive Health		Y	
560 = Midwifery (Available From October 1995)		Y	
600 = General Medical Practice		Y	
601 = General Dental Practice		Y	
610 = General Practice With Maternity Function (Available To 2003-04)		Y	
620 = General Practice Other Than Maternity (Available To 2003-04)		Y	
700 = Learning Disability (Previously Known As Mental Handicap)		Y	
710 = Adult Mental Illness		Y	
711 = Child And Adolescent Psychiatry			Y
712 = Forensic Psychiatry		Y	
713 = Psychotherapy		Y	
715 = Old Age Psychiatry (Available From 1990-91)		Y	
800 = Clinical Oncology (Previously Radiotherapy)		Y	
810 = Radiology		Y	
820 = General Pathology		Y	
821 = Blood Transfusion		Y	
822 = Chemical Pathology		Y	
823 = Haematology		Y	
824 = Histopathology		Y	
830 = Immunopathology		Y	
831 = Medical Microbiology And Virology		Y	
832 = Neuropathology (Available To 2003-04)		Y	
833 = Medical Microbiolody		Y	
834 = Medical Virology		Y	
900 = Community Medicine		Y	
901 = Occupational Medicine		Y	
902 = Community Health Services - Dental (Available From 2004-05)		Y	
903 = Public Health Medicine (Available From 2004-05)		Y	
904 = Public Health Dental (Available From 2004-05)		Y	
950 = Nursing Episode (Available From 2002-03)		Y	
960 = Allied Health Professional Episode (Available From 2006-07)		Y	

Supplementary material S3: Sensitivity analyses

Sampling timeframe

Healthcare use is known to vary with age. Age has the potential to be a confounder to the variable of interest (transition status) as those in adult care will generally be older than those in paediatric care. Sufficient years of data are needed to separate associations of the outcomes with age and with transition status. Inclusion of too many years decreases sample size (as it restricts to the models to individuals present for longer) and may underestimate short term associations with transition (effects of transition may be mostly short term, lasting a few years). Longer sampling timeframes may also introduce bias: for example, young people may change primary health care provider and leave the dataset around age 18 years if they move for education or employment and may differ from those who remain.

At least three years of data are needed, with at least two either in adult or paediatric healthcare to ensure age and transition status are not entirely collinear (over only two years of data, associations with age and transition would be indistinguishable). An a priori decision was made to use the last two years of paediatric data and first two years of adult data per cohort member and it was required that all cohort members were present in the data for at least these years. For the regressions, sensitivity analyses were conducted with all possible combinations of two, three and four years of paediatric data and one, two, three or four years of adult data.

Effects of sampling timeframe

Regressions were run with 2-4 years of data while in paediatric care and 1-4 years of data while in adult care.

Effects on incidence rate ratios dependent on transition status and age in year

Incidence rate ratios for the outcomes of emergency inpatient admissions and Emergency Department visits dependent on being in adult (compared to paediatric) healthcare and per year of age for males and females are shown in Figure S2, for the range of sampling timeframes tested.

The major differences are between regressions using one year of data when the young person was in adult healthcare or more than one year of data. Other variations in pre- and post-transition sampling years produce incidence rate ratios that have overlapping confidence intervals for the outcomes dependent on being in adult compared to paediatric care across all categories of condition and for the outcomes dependent on year of age except for the Emergency Department visits in the no long-term conditions group.

A key point is that when only one year of adult data is used, compared to those when more years of adult data are used, incidence rate ratios for the outcomes differ in opposing directions for being in adult (compared to paediatric) healthcare and age. This suggests a different model fit when only one year of adult data is used, with change sin outcomes being associated with changes in age rather than changes in healthcare. It is to be expected that too short a time period of data would make it more difficult to distinguish between associations with age and associations with transition status. This, and the apparent stability of incidence rate ratios once two or more years of adult data are used, suggests that it is the regressions using two or more years of data that most closely match the real associations between the outcomes, age and transition status.

Observations on model fit to all available data

Observed mean emergency inpatient admissions (Figure S3) and Emergency Department visits per person year (Figure S4) are plotted for the whole cohort using all data available from age 12 to 23 years, along with expected values from the regression models for the same population and age

range. It should be noted that the models were developed on a subset of these data: 2-4 years of data while in paediatric care and 1-4 years of data while in adult care, as indicated in the figures. Models and observations therefore sometimes diverge for ages for which there were few individuals included in the sample used to generate the models - for example, transition for young people with no long-term conditions was set to 16 years, so no young people were included when over 19 years of age (the largest sampling timeframe was four years of adult data, ending for this group at age 19 years).

Figures S3 and S4 demonstrate poor fit when only one year of adult data is used, particularly for emergency inpatient admissions (Figure 2). Differences for other sampling timeframes are much less marked, although there is observable worse fit for some of the lines using two years of paediatric data compared to three or four years of paediatric data (in particular, for emergency inpatient admissions for females with diabetes (Figure 2) and for Accident and Emergency visits for males with diabetes or no long-term conditions (Figure 3).

Appropriate choices of sampling time frame

Given the above, and the objectives set out in the main text regarding sampling timeframe selection, at least two years of paediatric and adult data appear to be required and the exact choice of sampling timeframe beyond that does not greatly change the conclusions drawn .

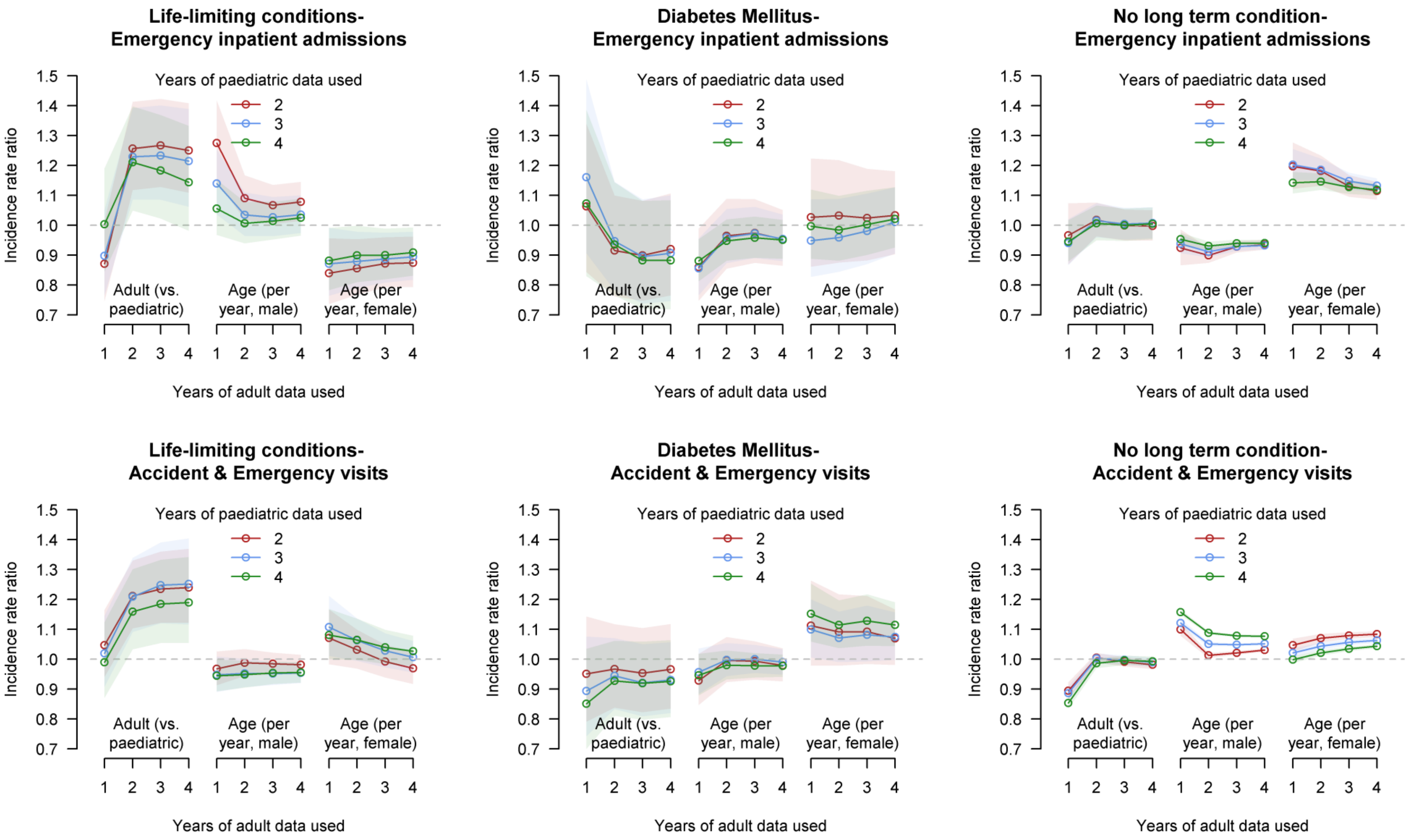

Figure S2: Incidence rate ratios for emergency inpatient admissions and Emergency Department visits associated with being in adult healthcare (versus paediatric healthcare) and per year of age for males and females, depending on the years of data pre- and post-transition used in the regressions. Shaded regions indicate 95% confidence intervals.

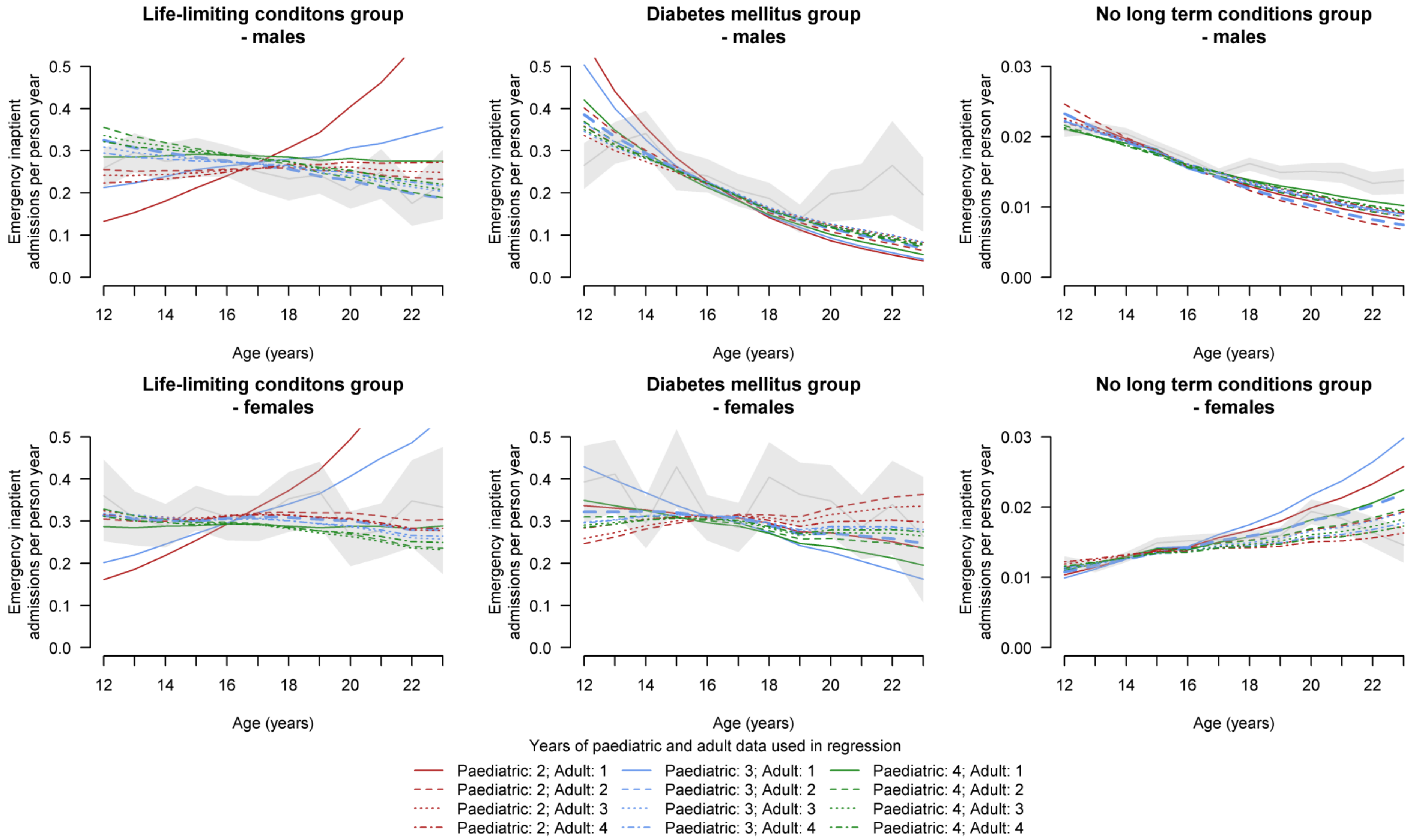

Figure S3: Fits of predicted numbers of emergency inpatient admissions per person per year by age from the models (with indicated years of data used) against all available data for cohort members.

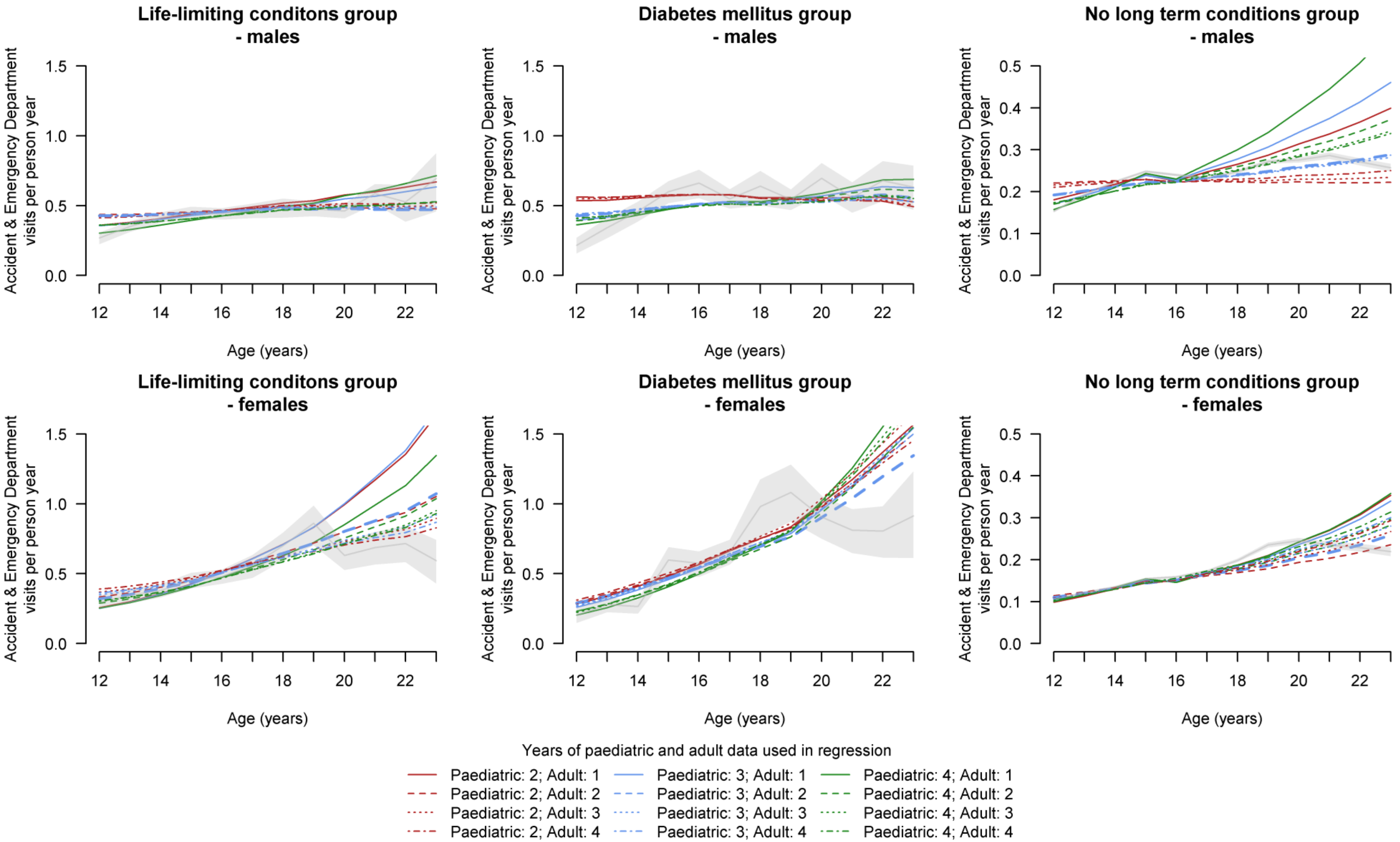

Figure S4: Fits of predicted numbers of Emergency Department visits per person per year by age from the models (with indicated years of data used) against all available data for cohort members.

Minimum age at exit from dataset required to have transition assigned

The results were insensitive to variations in the required minimum age at exit from the dataset required for transition to be set to 16 years in the absence of sufficient data to estimate transition (Table S3 and S4). In practice, few individuals were present for long enough to have data for the last two years of paediatric care and first two years of adult care with a transition age of 16 years (i.e. present from at least age 14-17 years) for these decisions to have much impact.

Table S3: Regression model incidence rate ratio for two level Poisson regressions on the numbers of emergency inpatient admissions and number of Emergency Department visits per cohort member per year when restrictions on age at exit from the dataset to have transition assigned are removed. *indicates there are omitted combinations of interactions (reference groups with incident rate ratio 1).

	Emergency inpatient admissions				Emergency Department visits			
	Incidence rate ratio	95% confidence interval		P value	Incidence rate ratio	95% confidence interval		P value
Age (per year of age)	0.90	0.87	0.93	<0.01	1.02	1.01	1.03	<0.01
**Sex**								
Male	1 (ref)				1 (ref)			
Female	0.06	0.03	0.12	<0.01	0.22	0.18	0.28	<0.01
**Condition group**								
No long-term condition	1 (ref)				1 (ref)			
Diabetes	16.03	2.12	121.21	0.01	3.12	0.80	12.13	0.10
Life-limiting condition	5.04	1.44	17.60	0.01	2.97	1.36	6.49	0.01
**Transition status**								
Paediatric care	1 (ref)				1 (ref)			
Adult care	1.00	0.95	1.06	0.95	1.01	0.99	1.02	0.43
**Sex × Age interaction***								
Female (per year of age)	1.19	1.13	1.24	<0.01	0.22	0.18	0.28	<0.01
**Condition group × Age interaction***								
Diabetes (per year of age)	0.99	0.88	1.12	0.93	0.98	0.91	1.06	0.70
Life-limiting conditions (per year of age)	1.08	1.00	1.16	0.05	0.98	0.93	1.03	0.35
**Condition group × Sex × Age interaction***								
Diabetes and female (per year of age)	1.01	0.85	1.20	0.92	1.12	0.99	1.26	0.07
Life-limiting conditions and female (per year of age)	0.85	0.75	0.96	0.01	1.04	0.97	1.11	0.31
**Condition group × Transition status interaction***								
Diabetes and adult care	0.82	0.67	1.01	0.07	0.93	0.82	1.06	0.30
Life-limiting condition and adult care	1.32	1.15	1.51	<0.01	1.23	1.11	1.37	<0.01

Table S4: Combined incidence rate ratios (taking account of interactions) for emergency inpatient admissions and Emergency Department visits when restrictions on age at exit from the dataset to have transition assigned are removed.

Group	Emergency inpatient admissions				Emergency Department visits			
	Incidence rate ratio (adult compared to paediatric care)	95% confidence interval		P value	Incidence rate ratio (adult compared to paediatric care)	95% confidence interval		P value
Life-limiting conditions	1.32	1.17	1.50	<0.01	1.24	1.12	1.37	<0.01
Diabetes	0.83	0.68	1.01	0.06	0.94	0.83	1.07	0.34
No long-term conditions	1.00	0.95	1.06	0.95	1.01	0.99	1.02	0.43

Exclusion of ethnic group and deprivation group

Inclusion or exclusion of ethnic group and deprivation group had little effect on incidence rate ratios for other variables in the models (Table S5 and S6). Ethnic group and deprivation group were both at level 1 (individual) and so were partly accounted for by the use of a random intercept in the models.

Table S5: Regression model incidence rate ratio for two level Poisson regressions on the numbers of emergency inpatient admissions and number of Emergency Department visits per cohort member per year when ethnic group and deprivation category are excluded from the models. *indicates there are omitted combinations of interactions (reference groups with incident rate ratio 1).

	Emergency inpatient admissions				Emergency Department visits			
	Incidence rate ratio	95% confidence interval		P value	Incidence rate ratio	95% confidence interval		P value
**Age (per year of age)**	0.90	0.87	0.93	<0.01	1.02	1.01	1.03	<0.01
**Sex**								
Male	1 (ref)				1 (ref)			
Female	0.06	0.03	0.12	<0.01	0.22	0.18	0.28	<0.01
**Condition group**								
No long-term condition	1 (ref)				1 (ref)			
Diabetes	16.16	2.14	121.97	0.01	3.16	0.81	12.29	0.10
Life-limiting condition	6.32	1.80	22.16	<0.01	3.39	1.54	7.47	<0.01
**Transition status**								
Paediatric care	1 (ref)				1 (ref)			
Adult care	1.00	0.95	1.06	0.95	1.01	0.99	1.02	0.43
**Sex × Age interaction***								
Female (per year of age)	1.19	1.13	1.24	<0.01	1.07	1.06	1.09	<0.01
**Condition group × Age interaction***								
Diabetes (per year of age)	0.99	0.88	1.12	0.92	0.98	0.91	1.06	0.69
Life-limiting conditions (per year of age)	1.07	0.99	1.15	0.10	0.97	0.92	1.02	0.26
**Condition group × Sex × Age interaction***								
Diabetes and female (per year of age)	1.01	0.85	1.20	0.92	1.12	0.99	1.26	0.07
Life-limiting conditions and female (per year of age)	0.85	0.76	0.96	0.01	1.04	0.96	1.11	0.33
**Condition group × Transition status interaction***								
Diabetes and adult care	0.82	0.67	1.01	0.07	0.93	0.82	1.06	0.30
Life-limiting condition and adult care	1.33	1.16	1.53	<0.01	1.24	1.12	1.37	<0.01

Table S6: Combined incidence rate ratios (taking account of interactions) for emergency inpatient admissions and Emergency Department visits when ethnic group and deprivation category are excluded from the models

Group	Emergency inpatient admissions				Emergency Department visits			
	Incidence rate ratio (adult compared to paediatric care)	95% confidence interval		P value	Incidence rate ratio (adult compared to paediatric care)	95% confidence interval		P value
Life-limiting conditions	1.33	1.18	1.51	<0.01	1.25	1.13	1.38	<0.01
Diabetes	0.83	0.68	1.01	0.06	0.94	0.83	1.07	0.35
No long-term conditions	1.00	0.95	1.06	0.95	1.01	0.99	1.02	0.43

Inclusion of year of birth

Inclusion or year of birth for possible cohort effects (e.g. a young person born in 1992 experiencing different care and transition or being at a different stage of condition at transition age to a young person born in 2001) had little effect on incidence rate ratios for other variables in the models and on the combined associations of the outcomes with transition for each condition group (Tables S7 and S8).

Table S7: Regression model incidence rate ratio for two level Poisson regressions on the numbers of emergency inpatient admissions and number of Emergency Department visits per cohort member per year when year f birth is included in the models. *indicates there are omitted combinations of interactions (reference groups with incident rate ratio 1).

	Emergency inpatient admissions				Emergency Department visits			
	Incidence rate ratio	95% confidence interval		P value	Incidence rate ratio	95% confidence interval		P value
**Age (per year of age)**	0.90	0.87	0.93	<0.01	1.02	1.01	1.03	<0.01
**Year of birth (per year)**	0.99	0.98	1.00	0.03	1.07	1.06	1.07	<0.01
**Sex**								
Male	1 (ref)				1 (ref)			
Female	0.90	0.87	0.93	<0.01	0.22	0.17	0.27	<0.01
**Condition group**								
No long-term condition	1 (ref)				1 (ref)			
Diabetes	15.95	2.12	119.93	0.01	3.42	0.87	13.45	0.08
Life-limiting condition	6.37	1.82	22.25	<0.01	2.82	1.30	6.13	0.01
**Transition status**								
Paediatric care	1 (ref)				1 (ref)			
Adult care	1.00	0.95	1.06	0.94	1.00	0.99	1.02	0.56
**Sex × Age interaction***								
Female (per year of age)	1.19	1.13	1.24	<0.01	1.07	1.06	1.09	<0.01
**Condition group × Age interaction***								
Diabetes (per year of age)	0.99	0.88	1.12	0.93	0.98	0.91	1.06	0.68
Life-limiting conditions (per year of age)	1.07	0.99	1.15	0.10	0.98	0.94	1.03	0.50
**Condition group × Sex × Age interaction***								
Diabetes and female (per year of age)	1.01	0.85	1.20	0.92	1.12	0.99	1.26	0.06
Life-limiting conditions and female (per year of age)	0.85	0.76	0.96	0.01	1.04	0.96	1.11	0.34
**Condition group × Transition status interaction***								
Diabetes and adult care	0.82	0.67	1.01	0.07	0.93	0.82	1.06	0.30
Life-limiting condition and adult care	1.33	1.16	1.53	<0.01	1.23	1.11	1.36	<0.01

Table S8: Combined incidence rate ratios (taking account of interactions) for emergency inpatient admissions and Emergency Department visits when year of birth is included in the models.

Group	Emergency inpatient admissions				Emergency Department visits			
	Incidence rate ratio (adult compared to paediatric care)	95% confidence interval		P value	Incidence rate ratio (adult compared to paediatric care)	95% confidence interval		P value
Life-limiting conditions	1.33	1.18	1.51	<0.01	1.23	1.12	1.36	<0.01
Diabetes	0.83	0.68	1.01	0.06	0.94	0.83	1.07	0.33
No long-term conditions	1.00	0.95	1.06	0.94	1.00	0.99	1.02	0.56

Supplementary material S4: Age group splits for population estimates

Table S9: Estimated population levels of excess emergency inpatient admissions and excess Emergency Department visits each year for young people aged 14 to 23 years with life-limiting conditions in England in their first two years of adult care.

Age group	Number with life-limiting conditions in England	% in first two years of adult healthcare	Number in first two years of adult healthcare	Emergency inpatient admissions			Emergency Department visits		
				Expected in paediatric healthcare	Expected in adult healthcare	Excess associated with transition	In paediatric healthcare	In adult healthcare	Excess associated with transition
14-17	12495	22.90657	2862	809	1044	235	1381	1719	338
18-23	19324	34.52585	6672	1785	2302	518	3524	4387	863
**14-23**	**31819**	**27.94478**	**9534**	**2594**	**3346**	**753**	**4905**	**6106**	**1201**
